# Effects of Pulse Parameters on Weld Microstructure and Mechanical Properties of Extra Pulse Current Aided Laser Welded 2219 Aluminum Alloy Joints

**DOI:** 10.3390/ma10091091

**Published:** 2017-09-15

**Authors:** Xinge Zhang, Liqun Li, Yanbin Chen, Zhaojun Yang, Yanli Chen, Xinjian Guo

**Affiliations:** 1 School of Mechanical Science and Engineering, Jilin University, Changchun 130025, China; yangzj@jlu.edu.cn (Z.Y.); chenyanli@jlu.edu.cn (Y.C.); 2 State Key Laboratory of Advanced Welding and Joining, Harbin Institute of Technology, Harbin 150001, China; lilqhit@163.com (L.L.); chenyanbinhit@163.com (Y.C.); xijguo@163.com (X.G.)

**Keywords:** laser welding, aluminum alloy, pulse current, weld microstructure, mechanical properties

## Abstract

In order to expand the application range of laser welding and improve weld quality, an extra pulse current was used to aid laser-welded 2219 aluminum alloy, and the effects of pulse current parameters on the weld microstructure and mechanical properties were investigated. The effect mechanisms of the pulse current interactions with the weld pool were evaluated. The results indicated that the coarse dendritic structure in the weld zone changed to a fine equiaxed structure using an extra pulse current, and the pulse parameters, including medium peak current, relatively high pulse frequency, and low pulse duty ratio benefited to improving the weld structure. The effect mechanisms of the pulse current were mainly ascribed to the magnetic pinch effect, thermal effect, and electromigration effect caused by the pulse current. The effect of the pulse parameters on the mechanical properties of welded joints were consistent with that of the weld microstructure. The tensile strength and elongation of the optimal pulse current-aided laser-welded joint increased by 16.4% and 105%, respectively, compared with autogenous laser welding.

## 1. Introduction

Owing to high energy density, the laser welding (LW) process provides the ability to produce the desired welds with a high depth-to-width ratio, narrow heat affected zone (HAZ), little distortion, high flexibility, and high welding speed. However, the shortcomings of the laser welding process involve the high price of the laser equipment, the instability of the process, and the probability of crack and pore defects, especially while laser welding highly-reflective material, such as aluminum alloy [1,2,3]. In past decades, for the purpose of the mitigation of the above problems, and wider application of laser welding, external electric or magnetic fields have been used to aid laser welding in the investigations. Kern et al. [4] put forward an electric current applied to the laser weld pool because of the thermoelectric voltage between the base metal and the melt in the weld pool; thus, the magnetically-supported laser beam welding process was developed. Lindenau et al. [5] and Vollertsen et al. [6] separately studied magnetically-supported laser-welded aluminum alloys, and the results showed that the top appearance and cross-section of the weld were indeed improved, and the degree of dilution increased due to the electromagnetic stirring effect. Xiao et al. [7,8,9] reported that by directly applying extra current to the weld pool of laser-welded aluminum alloy, the weld penetration and weld area all significantly increased, but other effects on the weld microstructure and mechanical properties had not been evaluated.

Previous publications stated that most investigations of the current-improving aluminum alloy solidification structures were carried out in the casting and arc-welding field. Crossley et al. [10] found that the solidification macrostructure of aluminum alloy became coarse with extra continuous current, which was attributed to a large amount of Joule heat generated by the extra continuous current. Zi et al. [11] studied the solidification structure of LY12 aluminum alloy under pulsed current with high density, and the microstructure was obviously refined and equiaxed. In addition, the stronger the density of the pulse current, the better the refining effect. Karunakaran et al. [12] reported that the grains in the fusion zone were refined using pulsed current tungsten inert gas (TIG) welding; as a result, the tensile properties and hardness of the welded joints increased. Cong et al. [13] welded aluminum alloy using an ultrafast-convert high-frequency pulse current TIG welding process, and the tensile strength and elongation of the weld joints increased 22%and 111%, respectively, compared with that of the conventional process. Potluri et al. [14] and Balasubramanian et al. [15] performed the investigation on metal inert gas (MIG) welding of aluminum alloy with pulsed current, and the mechanical properties were improved compared with continuous-current MIG welding.

2219 aluminum alloy is commonly used as rocket fuel tank material in aviation and space industries because of its high strength-to-weight ratio, high-temperature mechanical properties, and high corrosion resistance [16,17,18]. In recent years, TIG welding [19], MIG welding [20], electron beam welding [21], friction stir welding [22], and variable polarity plasma arc-welding [23], etc., were employed to weld 2219 aluminum alloy. However, there were rare reports of the laser welding of 2219 aluminum alloy. In this study, the bead-on-plate welded 2219 aluminum alloy plates were accomplished by extra pulse current-aided laser welding (PCALW). The effects of the pulse parameters on the weld microstructure and the effect mechanisms were evaluated. Furthermore, the mechanical properties, including microhardness, tensile strength, and elongation of the welded joints were investigated.

## 2. Materials and Methods

In the present investigation, 2219 aluminum alloy plates were used as the base metal (BM) with dimensions of 200 mm × 100 mm × 3.0 mm. The chemical composition of the base metal is specified in Table 1. Before welding, the aluminum alloy plates were professionally cleaned to remove impurities and oxides.

**Figure 1:**
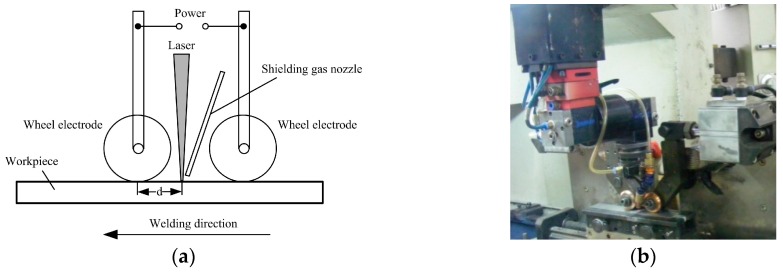
(**a**) Schematic diagram of extra pulse current aided laser welding; (**b**) experimental system.

**Figure 2:**
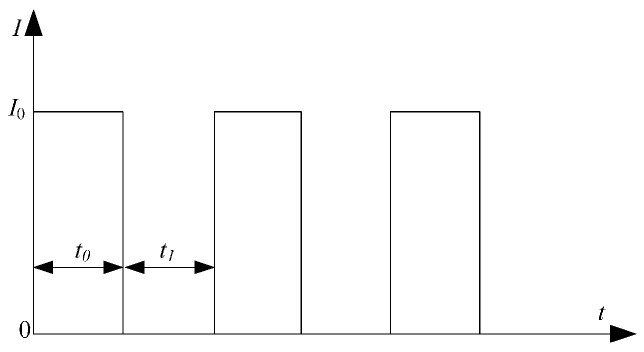
Schematic diagram of pulse current waveform.

Thebead-on-plate welding was performed using a CO_2_ laser (3 kW maximum power, DC030; Rofin-Sinar, GmbH, Hamburg, Germany) source and the extra pulse current was applied to the specimen by two wheel electrodes, as shown in Figure 1. The extra current was supplied by a combination device (PSG6130 and PSI6200; Bosch Rexroth Company China, Shanghai, China) which could vary from 2.0 kA to 13.0 kA. During the welding process, the laser beam was focused on the specimen with a 190 mm focal length lens and the spot diameter was 0.2 mm. Two wheel electrodes pressed on the top surface of the specimen and the laser beam located the center between two wheel electrodes. While extra current aided laser welding, the specimens moved with the traveling device. At the same time, the laser beam was fixed and two wheel electrodes rotated about a fixed axis. The distance “d” (shown in Figure 1) between the position of the wheel electrode bottom and the position of the laser beam focus was fixed at 25 mm. The shielding gas was pure argon, and the flow rate was 25 L/min. The laser power and welding velocity were kept constant at 2.9 kW and 0.6 m/min, respectively, with variable pulse current. The schematic diagram of the pulse current waveform is shown in Figure 2; *I*_0_ is the peak value of the pulse current and *t*_0_ is the time *I*_0_ was applied. The base value of the pulse current is zero and *t*_1_ is the time that the base value of current was applied. Therefore, the pulse frequency of current *f*_H_ is equal to 1/(*t*_0_ + *t*_1_) and the pulse duty ratio of current *δ* is equal to *t*_0_/(*t*_0_ + *t*_1_).

**Figure 3:**
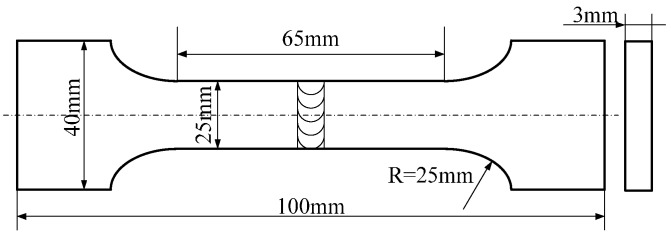
Dimensions of the tensile test specimen.

After the PCALW, the welded specimens were sectioned using a wire electrical-discharge cutting machine, mounted on 240#, 600#, 800#, 1500# grit SiC paper, and polished for metallographic examination. The metallographic observation samples were etched for 5 s using the solution of 1 vol % HCl, 1.5 vol % HF, 2.5 vol % HNO_3_, and 95 vol % H_2_O. The microstructure features were characterized by optical microscope (OM) (Eclipse E200, Nikon Instruments (Shanghai) Co., Ltd., Shanghai, China), and scanning electron microscopy (SEM) (S-4700, Hitachi High-Technologies in China, Suzhou, China) with an energy-dispersive X-ray spectrometer (EDS). The phase constitutions were identified by an X-ray diffractometer (XRD) (D/MAX-rB 12 KW, Rigaku, Tokyo, Japan) equipped with Cu K*α* (*λ* = 0.154045 nm) radiation. The XRD data were collected with a scanning speed of 4°/min and the 2*θ* was from 20° to 100°. The microhardness test was carried out using a microhardness testing machine (HVS-5, Zhuhai Precision Instrument Co., Ltd., Zhuhai, China) with a 150 g test load. The dimensions of the tensile test specimen are shown in Figure 3. The tensile test was conducted at room temperature using an Instron-5569 material tensile machine with a strain speed of 2 mm/min. The fracture surface of tensile specimens were examined using S-4700.

## 3. Results and Discussion

### 3.1. Effect of Extra Current onthe Weld Zone

**Figure 4:**
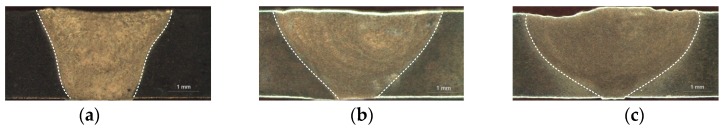
Typical PCALW morphology of (**a**) *I*_0_ = 0, autogenous laser welding; (**b**) *I*_0_ = 4 kA; and (**c**) *I*_0_ = 8 kA.

Figure 4 shows the typical weld morphology of welded specimens produced with different extra current-aided laser welding of 2219 aluminum alloy with fixed process parameters. It can be observed that the weld area and top weld width of the PCALW joint obviously increase, but the bottom weld width reduces compared with that of laser welding. During the PCALW process, the current density in the upper part of the weld pool was higher than the lower part owing to the skin effect of the current, causing an accelerating fluid flow along the width direction of the weld pool. This resulted in the increase of the upper part of the weld and the evident decrease of the lower part of the weld. While the extra current increased up to 8 kA, the bottom weld width reduced to a very small size. In order to obtain welded joints with full penetration, the maximum value of the current used in this systematic experimental investigation was 8 kA in this study.

**Figure 5:**
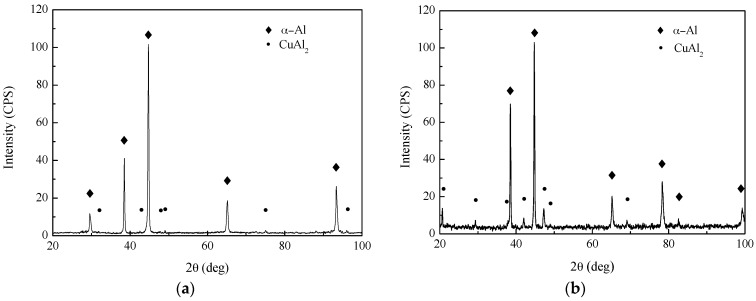

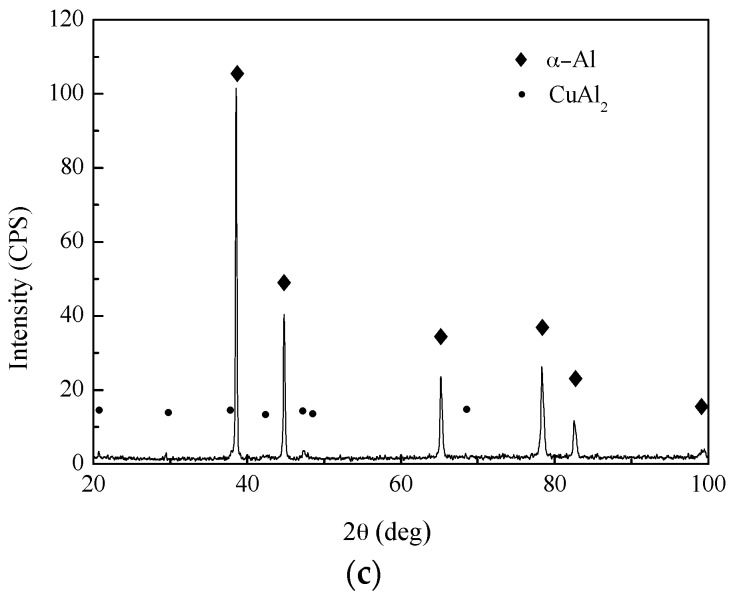
XRD patterns of (**a**) base metal; (**b**) weld metal of laser welding; and (**c**) weld metal of PCALW.

**Figure 6:**
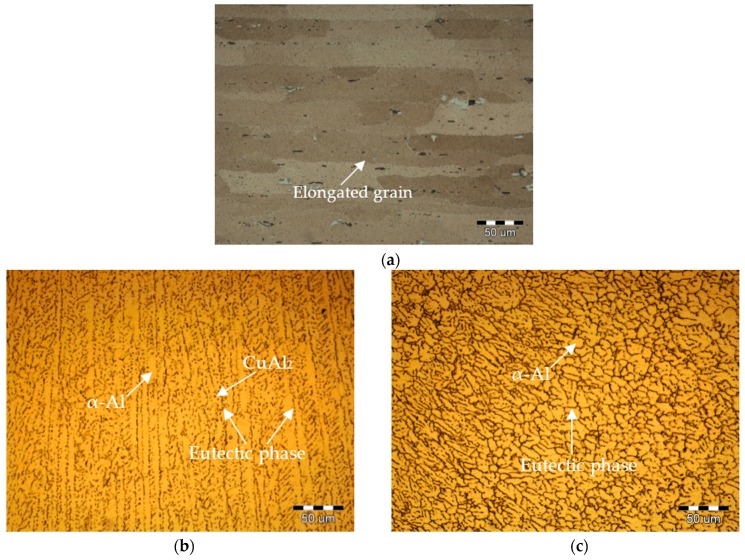
Microstructure of (**a**) base metal; (**b**) weld of laser welding; and (**c**) weld of PCALW.

Figure 5 illustrates the XRD patterns of base metal and weld metal. All of the diffraction patterns show the presence of *α*-Al phase main peaks and a certain number of CuAl_2_ (*θ* phase) peaks without other phase peak due to the small amount. The CuAl_2_ phase peak intensity in the weld metal is higher than that of the base metal. In comparison with laser welding, the CuAl_2_ phase peak intensity of the PCALW weld metal decreases evidently, owing to the suppressed eutectic reaction by the effect of the extra pulse current. The microstructures of the base metal, weld zone of the laser welding, and PCALW are indicated in Figure 6. It can be seen that the microstructure of the base metal is composed of elongated grains with irregularly distributed precipitates, which include CuAl_2_ and a small amount of Cu-Mn-Al compound, as shown in Figure 6a. The microstructure of the weld zone of laser welding mainly consists of a coarse dendritic structure and the *α* + *θ* eutectic phase distributes within the grain boundary. Meanwhile, a large amount of CuAl_2_ precipitates exist within the dendritic grains. With an extra pulse current, the coarse structure in the weld zone changed to a fine equiaxed structure, and there were few CuAl_2_ precipitates within the equiaxed structure. The microstructure observations were consistent with the XRD analysis results in Figure 5.

**Figure 7:**
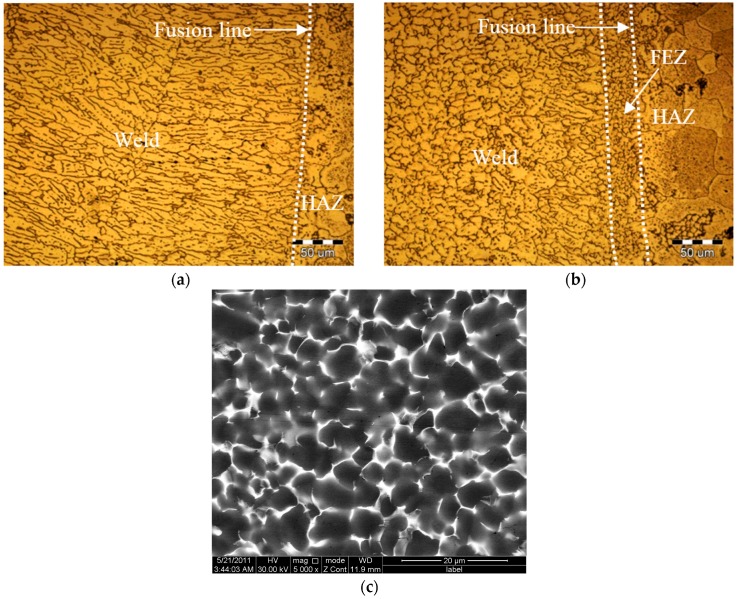
Microstructure of (**a**) autogenous laser welding; (**b**) PCALW; and (**c**) a SEM image of the FEZ.

Another obvious observation is that there is a fine equiaxed zone (FEZ) near the fusion line for PCALW joint, but the columnar dendritic structures grow along perpendicular direction to the fusion line of the autogenous laser-welded joint, as shown in Figure 7. Figure 7c is the SEM image at 5000× magnification of the FEZ shown in Figure 7b, and it indicates that the eutectic phase homogeneously distributes along the grain boundaries, and the fine grains are subglobose or polygonal, as shown in Figure 7c. Meanwhile, there are no separating phases within the grains. The width of the FEZ is narrow, about 30 μm, and the grains are fine, which were all beneficial to improve the mechanical properties near the fusion line for the aluminum alloy welded joint.

### 3.2. Effect of Extra Pulse Current Parametersonthe Weld Microstructure

With extra pulse current-aided laser welding of 2219 aluminum alloy, the extra pulse current parameters mainly included pulse peak current *I*_0_, pulse frequency *f*_H_, and pulse duty ratio *δ*. The effects of every single parameter of the pulse current on weld microstructure with the other process parameters fixed were investigated in detail.

#### 3.2.1. Effect of Peak Current *I*_0_ on the Weld Microstructure

**Figure 8:**
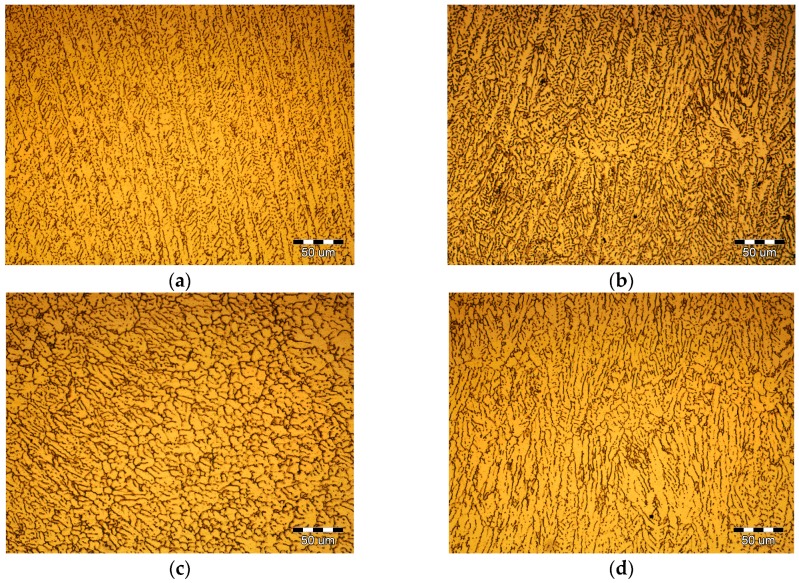
Effect of peak current *I*_0_ on the PCALW weld microstructure for: (**a**) *I*_0_ = 0 (autogenous laser welding); (**b**) *I*_0_ = 4 kA; (**c**) *I*_0_ = 6 kA; and (**d**) *I*_0_ = 8 kA.

Figure 8 shows the PCALW weld microstructure with different peak current *I*_0_. The *t*_0_ and *t*_1_ were 10 ms and 20 ms; namely, the pulse frequency *f*_H_ and pulse duty ratio *δ* were 100/3 Hz and 1/3, respectively. The coarse dendritic structure was improved until the peak current *I*_0_ reached 4 kA and a small amount of the equiaxed dendritic structure grew in the weld center, as shown in Figure 8b. When the increasing peak current *I*_0_ to 6 kA, the structure was obviously refined, which consisted of an equiaxed dendritic structure and a subglobose equiaxed non-dendritic structure, as shown in Figure 8c. However, when the peak current *I*_0_ increases to 8 kA, the coarse dendritic structures appear again, illustrated in Figure 8d. Therefore, if the pulse frequency *f*_H_ and pulse duty ratio *δ* were constant, there was a maximum peak current *I*_0_ which could be used to obtain the optimal weld structure.

#### 3.2.2. Effect of the Pulse Frequency *f*_H_ on the Weld Microstructure

**Figure 9:**
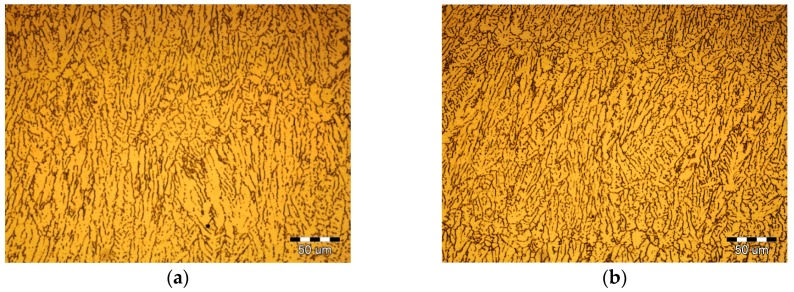

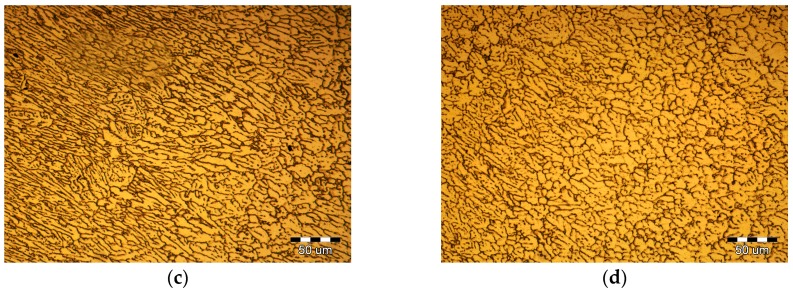
Effect of pulse frequency *f*_H_ on the PCALW weld microstructure for: (**a**) *f*_H_ = 5/3 Hz; (**b**) *f*_H_ = 10/3 Hz; (**c**) *f*_H_ = 20/3 Hz; and (**d**) *f*_H_ = 100/3 Hz.

Figure 9 shows the PCALW weld microstructure with different pulse frequency *f*_H_. The pulse duty ratio *δ* was 1/3, therefore, the *t*_0_ and *t*_1_ were assigned as: 200 and 400 ms, 100 and 200 ms, 50 and 100 ms, and 10 and 20 ms; namely, the pulse frequency *f*_H_ values were 5/3, 10/3, 20/3 Hz, and 100/3 Hz, respectively. The peak current *I*_0_ was 6 kA. The results clearly indicate that the coarse dendritic structure developed in the weld with lower pulse frequency *f*_H_, as shown in Figure 9a,b. While the pulse frequency *f*_H_ increases to 20/3 Hz, the structure is refined, which mainly consists of a columnar dendritic structure, and the equiaxed non-dendritic structure appears, shown in Figure 9c. To continue to increase pulse frequency *f*_H_, the columnar dendritic structure transforms to equiaxed dendritic and non-dendritic structures, shown in Figure 9d. Thence, the high pulse frequency *f*_H_ was beneficial to improving the weld structure.

#### 3.2.3. Effect of the Pulse Duty Ratio *δ* on the Weld Microstructure

**Figure 10:**
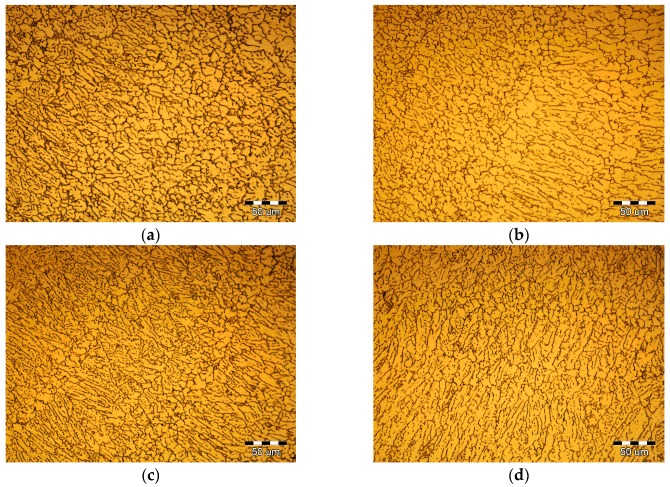
Effect of the pulse duty ratio *δ* on the PCALW weld microstructure for: (**a**) *δ* = 1/3; (**b**) *δ* = 1/2; (**c**) *δ* = 2/3; and (**d**) *δ* = 1.

Figure 10 shows the PCALW weld microstructure with different pulse duty ratio *δ*. The pulse frequency *f*_H_ was 100/3 Hz, so, the *t*_0_ and *t*_1_ were assigned as: 10 and 20 ms, 15 and 15 ms, 20 and 10 ms, 30 and 0 ms; namely, the pulse duty ratio *δ* values were 1/3, 1/2, 2/3, and 1, respectively. The peak current *I*_0_ was also 6 kA. Comparing with autogenous laser welding, the PCALW weld structures are all refined in Figure 10, which is attributed to the optimal peak current *I*_0_ and pulse frequency *f*_H_. The lower pulse duty ratio *δ* leads to a fine equiaxed structure shown in Figure 9a. With the increase of the pulse duty ratio *δ*, the number of equiaxed non-dendritic structures gradually decreased. When the pulse duty ratio *δ* is 1, namely, an applied continuous current, the growth direction of columnar dendritic structuresare distinct and the eutectic phase grew, which distributed along the coarse grain boundaries, as shown in Figure 10d. Thus, it is favorable to choose a low pulse duty ratio *δ* for PCALW of 2219 aluminum alloy.

As a consequence of the above results, the favorable pulse current parameters, including the medium peak current *I*_0_, relatively high pulse frequency *f*_H_, and low pulse duty ratio *δ*, can be chosen to improve the weld microstructure of laser-welded 2219 aluminum alloy.

### 3.3. Discussion of Effect Mechanism of the Extra Pulse Current

Several investigations have been reported in the literature on the solidification structure improved using a pulse current [11,12,13,14,15]. However, there is still no generally unified theory of effect mechanism of pulse current on the solidification structure at present. In this study, the magnetic pinch effect, Joule thermal effect, and electromigration effect caused by the pulse current will be individually analyzed and discussed qualitatively.

#### 3.3.1. Magnetic Pinch Effect

On the basis of Maxwell’s equations, while the pulse current passes through the melting alloy, a pulsed magnetic field is produced, which interacts with the pulse current and results in a magnetic pinch force. The magnetic pinch force will influence the melting alloy. Yan. [24] put forward that the electromagnetic force per unit area P could be described by the following equation:

(1) $$P=\frac{\mu_{0}j^{2}r^{2}}{8\pi^{2}R^{4}}$$

where *P* is the electromagnetic force per unit area, $\mu_{0}$ is the permeability, *j* is the current density, *r* is the distance between any point and the center line, and *R* is the sectional radius of the melt.

From Equation (1), it can be seen that the *P* will increase with the increase of *j*, while *P* is greater than the momentum pressure, which causes a periodic extrusion to the melting alloy in the weld pool. The periodic extrusion induced by the magnetic pinch effect should cause the molten pool to rapidly lose overheating and reduce the degree of super cooling, which affects the rate of nucleation. The rate of nucleation *N* can be determined by the following equations [24]:

(2) $$\ln N=\ln a_{1}-\frac{a_{3}}{{\left(a_{4}+P\right)}^{2}}-a_{2}P$$

and $a_{1}=\left[\frac{nKT}{h}\right]\exp\left(-fe^{\delta /T}\right)$; $a_{2}=f\beta \exp\left(\delta /T\right)$; $a_{3}=\frac{16\pi \sigma_{\mathrm{SL}}{}^{3}T_{m}{}^{3}}{3KTL_{m}{}^{2}\alpha^{2}}$; $a_{4}=\Delta T_{0}/\alpha$, where *P* is the electromagnetic force per unit area; *n* is atom number per unit area; *K* is Boltzman’s constant; *T* is the temperature; *h* is Planck’s constant; *f*, *δ*, and *β* are constant coefficients; $\sigma_{\mathrm{SL}}$ is the surface free energy per unit; $T_{m}$ is the melting point; $L_{m}$ is the latent heat; $\Delta T_{0}$ is the degree of super cooling; and *α* is a constant, dependent on material.

According to Equation (2), while *P* is at a low level, the rate of nucleation *N* increases as *P* increases; while *P* is at a high level, the rate of nucleation *N* reduces as *P* increases. In other words, the rate of nucleation *N* can reach a maximal value with an optional *P*. Since *P* is directly proportional to current density *j* from Equation (1), the rate of nucleation *N* can reach a maximum with an optional *j* depending upon the peak current *I*_0_.

Hunt [25] reported that the transition from the columnar structure to the equiaxed structure required the following condition:

(3) $$G_{L}＜0.061N^{1/3}\left[1-{\left(\Delta T_{N}\right)}^{3}/{\left(\Delta T_{C}\right)}^{3}\right]\Delta T_{C}$$

where $G_{L}$ is the liquid temperature gradient at the solid-liquid interface; *N* is the rate of nucleation; atom number per unit area; $\Delta T_{N}$ is the critical degree of supercooling for nucleation; $\Delta T_{C}$ is the degree of supercooling at the front of the columnar structure. Equation (3) demonstrates that the increase in the rate of nucleation *N* will promote the transition from the columnar structure to the equiaxed structure during melting alloy solidification. Thus, there was a maximum peak current *I*_0_ used to obtain optimal weld structure during the PCALW process, as shown in Figure 8.

During the solidification of the melting alloy in the weld pool, the periodic extrusion could also make the dendritic eutectic phase fragment, which would suppress the growth of eutectic phase and then the fragmented crystals would become new crystal nuclei which help to produce the fine equiaxed structure. The smaller the pulse frequency *f*_H_, the better the fragmented crystal effect, and the more benefit to improving the weld structure, as shown in Figure 9.

#### 3.3.2. Thermal Effect

When extra current passes through the weld pool, several thermal effects, such as Joule heating, and thermoelectric effects [26]. Qin [27] introduced that the Joule heating generated by the extra pulse current had a great effect on the rate of nucleation, and the relation between the rate of nucleation N and pulse current was expressed as:

(4) $$N=A\exp\left(-\left(\frac{16\pi \sigma_{\mathrm{sl}}{}^{3}T_{m}{}^{2}}{3L_{m}{}^{2}\Delta T^{2}}+\Delta G_{A}\right)/K\left(T+j^{2}\rho_{e}t/\rho c\right)\right)$$

where *A* is the constant independent of temperature; $\sigma_{\mathrm{SL}}$ is the surface free energy per unit; $T_{m}$ is the melting point; $L_{m}$ is the latent heat; ΔT is the latent heat; $\Delta G_{A}$ is the diffusion activation energy; *K* is Boltzman’s constant; *T* is the temperature; j is the current density, $\rho_{e}$ is the resistivity; *t* is the time of the current pass per pulse (*t*_0_ in this study, shown in Figure 2); and ρ and c are the density and heat enthalpy, respectively.

According to Equation (4), while the value of current density j is fixed, the rate of nucleation *N* decreases with the increase of *t* and the *t* is equal to *t*_0_, as shown in Figure 2. During the PCALW process, while the pulse frequency *f*_H_ was fixed, and the *t*_0_ increases with the increase of the pulse duty ratio *δ*. Therefore, the rate of nucleation *N* would decrease with the increase of the pulse duty ratio *δ*. Then, from the Equations (3) and (4), it can be found that the lower pulse duty ratio *δ* are more help to refine weld structure, which is consistent with the weld microstructure observation result in Figure 10.

During the solidification process of the melting alloy in the weld pool, owing to the difference of electric resistance between the liquid eutectic phase and the solid Al matrix, when the extra current flowed in the liquid eutectic phase and the solid Al matrix, extra heat was generated at the interface between the liquid eutectic phase and the solid Al matrix caused by thermoelectric effects, which resulted in increasing the temperature of the liquid eutectic phase [26]. Therefore, the wettability was obviously improved between the Al matrix grain boundaries and the liquid eutectic phase, which brought about the transition from incomplete to complete grain boundary wetting. Finally, the eutectic phase uniformity distributed along grain boundaries in the weld zone with the extra pulse current.

#### 3.3.3. Electromigration Effect

**Figure 11:**
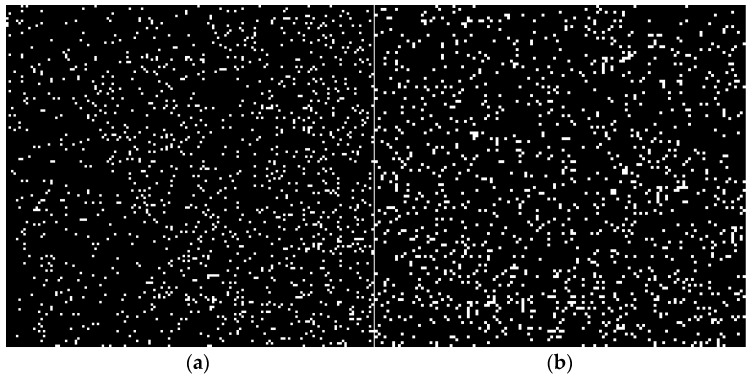
Distribution of Cu in the weld zone of (**a**) autogenous laser welding; and (**b**) PCALW.

It is well known that a large amount of metal ions exist within the liquid melting alloy. While the pulse current passes through the melting alloy, the metal ions will migrate under the electric field force. When the charge and quality of metal ion are *q* and *m*, respectively, the acceleration *a* induced by the electric field force can be described as:

(5) $$a=\frac{qj}{\rho_{e}m}$$

where *q* is the charge of the ion; *j* is the current density; $\rho_{e}$ is the resistivity; and *m* is the quality of the ion. From Equation (5), the acceleration *a* induced by the electric field force depends not upon the ratio of charge of ion *q* to quality of ion m. The *q*/*m* ratio of the Al ion is 3.5 times that of the Cu ion, namely, the acceleration *a* induced by the electric field force of the Al ion is 3.5 times that of Cu ion, which results in relative motion between the Cu ion (solute) and the Al ion (matrix). The Cu solute diffusion was greatly enhanced. Figure 11 displays the Cu element (white spot in Figure 11) distribution in the weld zone compared to autogenous laser welding with PCALW. It can be confirmed that the Cu solute in the PCALW weld zone is more uniformly distributed than that of autogenous laser welding. Due to the Cu solute, a more uniform distribution is induced by the electromigration effect, and the liquid metal solidification tended to homogeneous nucleation in the weld pool and the eutectic reaction was suppressed, which was considerably beneficial to the refined structure in the PCALW weld zone.

### 3.4. Mechanical Properties

#### 3.4.1. Microhardness Distribution

**Figure 12:**
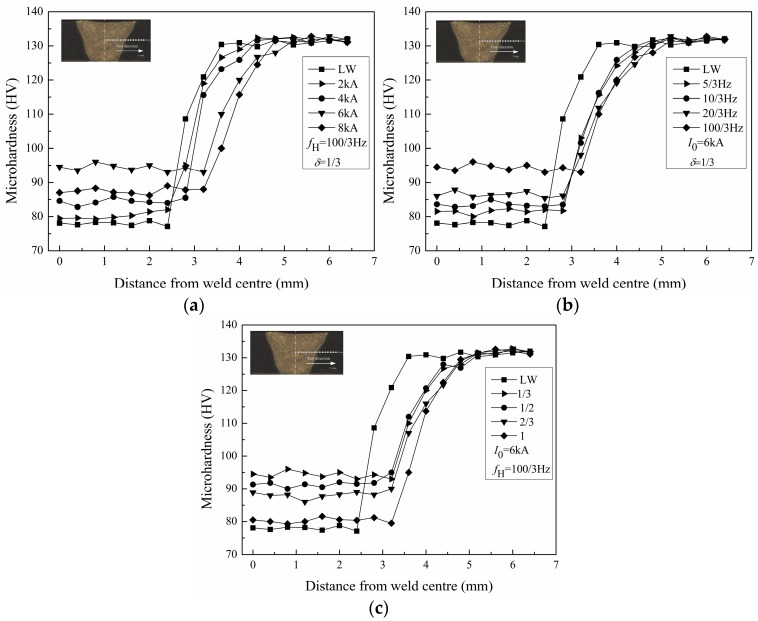
Microhardness distribution of welded joints with (**a**) different peak current *I*_0_; (**b**) different pulse frequency *f*_H_; and (**c**) different pulse duty ratio *δ*.

The microhardness was tested on the cross-sections of the welded joints along the middle of the plate thickness. The microhardness distributions of the welded joints with different pulse current parameters are shown in Figure 12. Figure 12 distinctly indicates that the microhardness in the weld zone is the lowest and the microhardness of the base metal is higher than that of the HAZ for all welded joints. In contrast with autogenous laser welding, the microhardness in the PCALW weld zone all increase, owing to the refined solidification structure.

Figure 12a reveals the effect of peak current *I*_0_ on microhardness distributions of the welded joint, and while the peak current *I*_0_ is 6 kA (intermediate value of peak current *I*_0_ in this study), the microhardness in the PCALW weld zone is highest. Figure 12b shows the effect of pulse frequency *f*_H_ on microhardness distributions of welded joint, and while the pulse frequency *f*_H_ is 100/3 Hz (maximum value of pulse frequency *f*_H_ in this study), the microhardness in the PCALW weld zone is the highest. Figure 12c displays the effect of the pulse duty ratio *δ* on the microhardness distributions of the welded joint, and while the pulse duty ratio *δ* is 1/3 (the minimum value of the pulse duty ratio *δ* in this study), the microhardness in the PCALW weld zone is the highest. The high microhardness in the weld zone is attributed to the refined solidification structure and the uniformly distributed Cu solute; therefore, the effects of the pulse current parameters on the microhardness in the weld zone accord with that of the microstructure.

#### 3.4.2. Tensile Properties

**Figure 13:**
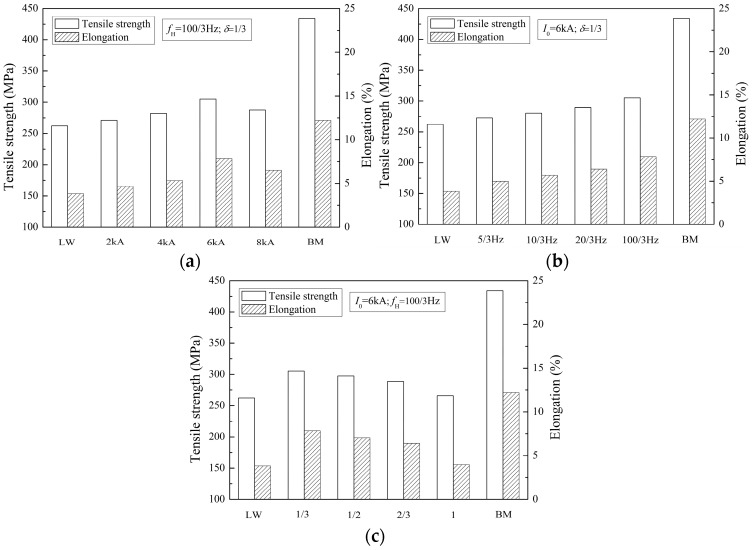
Tensile properties of welded joints with (**a**) different peak current *I*_0_; (**b**) different pulse frequency *f*_H_; and (**c**) different pulse duty ratio *δ*.

The tensile properties of the welded joints with different pulse current parameters are presented in Figure 13. The tensile strength and elongation of 2219 aluminum alloy are 434 MPa and 12.2%, respectively, which is a high-strength aluminum alloy. The tensile properties of all welded joints are lower than the base metal, however, the tensile properties of PCALW joints increases in contrast with autogenous laser welding, as shown in Figure 13. The optimal tensile strength and elongation of PCALW joints are 305.2 MPa and 7.83%, which are 70.3% and 64.2% of that of the base metal, respectively. It can be found that the tensile strength raises by 16.4% and the elongation raises by 105% using the optimal pulse current parameters (*I*_0_ = 6 kA, *f*_H_ = 100/3 Hz, *δ* = 1/3). The better tensile properties attributes to the improvements of the weld microstructure by extra pulse current. Hence, the effects of pulse current parameters (*I*_0_, *f*_H_, *δ*) on tensile properties of PCALW welded joints are the same as the effects on the weld microstructure.

**Figure 14:**
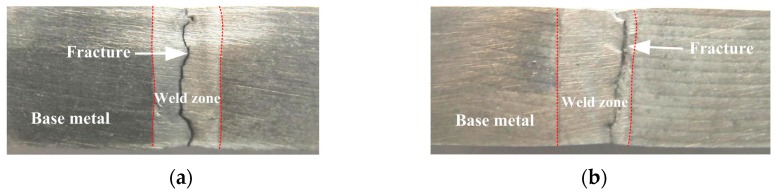
Fracture tensile samples of (**a**) autogenous laser welding; and (**b**) PCALW.

**Figure 15:**
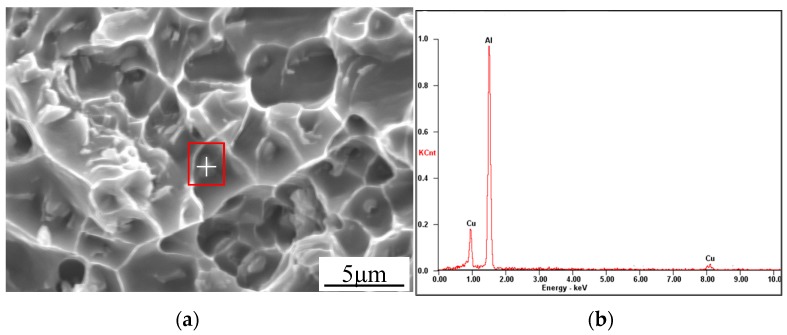
(**a**) Fracture surface morphology of the PCALW welded joint; and (**b**) EDS results of the particle.

During the tensile test, because of the lower microhardness than the HAZ and base metal shown in Figure 12, all of the welded joints rupture in the weld zone, as illustrated in Figure 14. Figure 14a indicates that the autogenous laser welded joint ruptures in the middle of weld zone owing to the coarse columnar dendritic structure with a distinct growth direction, shown in Figure 6b and Figure 7a. Figure 14b indicates that the PCALW welded joint with maximum tensile strength ruptures in the weld zone adjacent of the fine equiaxed zone, resulting from the relatively uneven distribution structure shown in Figure 6c and Figure 7b. The fracture surface morphology of the PCALW joint using SEM observations is displayed in Figure 15a, and it shows a typical dimple fracture feature. A large amount of particles was observed in the dimple, and EDS analysis of the particle was executed. The EDS results indicate that the particle consists of Al and Cu elements, as shown in Figure 15b, namely *α*-Al+CuAl_2_ eutectic phases, which are concordant with the above XRD analysis and microstructure observation results.

## 4. Conclusions

In the present paper, the effects of the pulse parameters on the weld microstructure and mechanical properties of extra pulse current-aided laser-welded 2219 aluminum alloy were investigated. Furthermore, the effect mechanisms of the extra pulse current interactions with the weld pool were evaluated. The main conclusions are summarized as follows:

(1) Using extra pulse current-aided laser welding, the coarse dendritic structure in the weld zone changed to the fine equiaxed structure, and the *α* + *θ* eutectic phase homogeneously distributed along the grain boundaries. A fine equiaxed zone existed in the PCALW joint in the vicinity of the fusion line, where the columnar dendritic structure grew perpendicularly for the autogenous laser welded joint.
(2) The favorable extra pulse current parameters, including medium peak current *I*_0_, relatively high pulse frequency *f*_H_, and low pulse duty ratio *δ*, can be chosen to refine the weld structure. The effective mechanisms of the extra pulse current are mainly ascribed to the magnetic pinch effect, thermal effect, and electromigration effect caused by the pulse current.
(3) The effect of pulse parameters on the mechanical properties of the welded joints were consistent with that of the weld microstructure. The microhardness in the weld zone was the lowest, and the laser-welded joint ruptured in the middle of weld zone, then, the PCALW welded joint with a maximum tensile strength ruptured in the weld zone adjacent to the fine equiaxed zone. The tensile strength and elongation of the optimal PCALW welded joint increased by 16.4% and 105% compared with autogenous laser welding.

## Figures and Tables

**Table 1:** Chemical composition of 2219 aluminum alloy (wt %).

Component	Cu	Si	Mn	Fe	Zr	Ti	Al
wt %	6.48	0.49	0.32	0.23	0.2	0.06	Balance
